# A predictive model for treatment response in patients with locally advanced esophageal squamous cell carcinoma after concurrent chemoradiotherapy: based on SUVmean and NLR

**DOI:** 10.1186/s12885-020-07040-8

**Published:** 2020-06-10

**Authors:** Chunsheng Wang, Kewei Zhao, Shanliang Hu, Yong Huang, Li Ma, Yipeng Song, Minghuan Li

**Affiliations:** 1 Department of Radiation Oncology, Qingdao University Medical College Affiliated Yantai Yuhuangding Hospital, 20 Yudong Road, Yantai, 264000 Shandong People’s Republic of China; 2 Department of Radiation Oncology, Shandong Cancer Hospital and Institute, Shandong University, 440 Jiyan Road, Jinan, 250117 Shandong People’s Republic of China; 3 Department of Nuclear Medicine, Shandong Cancer Hospital and Institute, Shandong University, 440 Jiyan Road, Jinan, 250117 Shandong People’s Republic of China

**Keywords:** Esophageal squamous cell carcinoma, Predictive model, Treatment response, Concurrent chemoradiotherapy, SUVmean, NLR

## Abstract

**Background:**

We conducted this study to combine the mean standardized uptake value (SUVmean) and neutrophil to lymphocyte ratio (NLR) to establish a strong predictive model for patients with esophageal squamous cell carcinoma (ESCC) after concurrent chemoradiotherapy (CCRT).

**Methods:**

We retrospectively analyzed 163 newly diagnosed ESCC patients treated with CCRT. Eighty patients (training set) were randomly selected to generate cut-off SUVmean and NLR values by receiver operating characteristic (ROC) curve analysis and to establish a predictive model by using the independent predictors of treatment outcomes. Then, we evaluated the performance of the prediction model regarding treatment outcomes in the testing set (*n* = 83) and in all sets.

**Results:**

A high SUVmean (> 5.81) and high NLR (> 2.42) at diagnosis were associated with unfavorable treatment outcomes in patients with ESCC. The prediction model had a better performance than the simple parameters (*p* < 0.05). With a cut-off value of 0.77, the prediction model significantly improved the specificity and positive predictive value for treatment response (88.9 and 92.1% in the training set, 95.8 and 97.1% in the testing set, and 92.2 and 91.8% in all sets, respectively).

**Conclusions:**

The pretreatment SUVmean and NLR were independent predictors of treatment response in ESCC patients treated with CCRT. The predictive model was constructed based on these two parameters and provides a highly accurate tool for predicting patient outcomes.

## Background

Concurrent chemoradiotherapy (CCRT) has been established as the standard treatment for locally advanced inoperable esophageal cancer (EC) patients, according to the phase III intergroup RTOG 85–01 trial [1]. Although CCRT improved local control and overall survival compared with radiotherapy alone, the treatment outcomes of CCRT varied widely. According to data in the literature, the overall response rate (ORR) to CCRT in patients with esophageal cancer ranges from 53.3 to 98.3% [2–4]. We can improve this rate by setting individualized treatment strategies and intensities for different subgroups of patients. However, it is quite difficult to balance the risks of complications and treatment benefits without knowing the effects before treatment. Therefore, the early prediction of the tumor response before treatment may benefit this heterogeneous group of patients.

^18^F-fluorodeoxy-glucose Positron emission tomography/computed tomography (^18^F-FDG PET/CT) allows visualization of the high glucose utilization in tumor tissue, based on the assumption that cancer cells generally exhibit a higher level of glycolytic activity than healthy cells. A semiquantitative parameter derived from FDG-PET, maximum standardized uptake values (SUVmax), has been widely used to quantitate the metabolic activity of tumors [5–7]. However, SUVmax is measured on a single voxel and may not reflect the metabolism within the whole tumor [8, 9]. Mean of standardized uptake values (SUVmean), another metabolic parameter, is subsequently measured to calculate the average SUVs above a threshold (SUV >  2.5), which might reflect the metabolic burden of the entire tumor as opposed to that of a single point [10, 11]. Previous studies on different solid tumors have shown a correlation between SUVmean and tumor treatment outcomes [12–14]. On the other hand, recent studies have revealed that cancer-related inflammation plays an important role in cancer progression and metastasis [15–17]. Neutrophil-to-lymphocyte ratio (NLR), as a systemic inflammatory marker, has been reported to be associated with tumor response and prognosis in esophageal cancer [18, 19]. However, these studies mainly explored the predictive effect of NLR in patients undergoing surgery, researches focused on the role of NLR in predicting tumor response in non-surgically patients have been rarely reported [20, 21].

Hence, in the present study, we attempted to establish a prediction model for the treatment effects of CCRT for esophageal cancer patients based on two aspects: the abnormal glucose metabolism of tumor cells and the anti-tumor immune response of the host.

## Methods

### Patients

We retrospectively analyzed 163 locally advanced ESCC patients who were treated with CCRT in shandong cancer hospital between January 2011 to December 2017. Patients were included if they had a Karnofsky performance scale (KPS) score ≥ 70 and had ESCC confirmed by histopathological analysis. They also need fulfilled the following criteria: (1) available complete clinical information;(2) completed PET/CT examination and routine blood test one week before any treatment;(3) No history of other malignancy or secondary primary tumor;(4) without any acute infections or any hematologic disease and autoimmune diseases; (5) locally advanced disease based on the 7th edition of the American Joint Committee on Cancer guidelines (AJCC7th edition). Of the 163 patients, 80 patients were randomly assigned to the training set using a computer program, while the remaining patients (*n* = 83) were assigned to the testing set. The ethics committee of Shandong Cancer Hospital and Institute approved the study. And informed consent was exempted due to the retrospective nature of the study.

### Treatment protocols and response assessment

All patients received intensity-modulated radiation therapy with a total dose of 50–64 Gy administered once daily with a standard fractionation (ie 1.8 or 2.0 Gy/ fractions, 25–32 fractions,5 days/week). Chemotherapy was administered simultaneously with the initial radiotherapy on Day 1. cisplatin (75 mg/m2) was administered by iv on Day 1 and 5-Fluorouracil (700 mg/m2) was administered intravenously (iv) continuous infusion over 24 h daily on Days 1–4. Cycled every 28 days for 2–4 cycles for 2 cycles with radiation followed by 2 cycles without radiation. Patients were asked to visit the clinic within 2–4 weeks after completion of all therapies. Contrast-enhanced computed tomography scan was performed 2–4 weeks after the end of treatment for evaluate treatment response. The tumor response was assessment based on evaluation criteria in solid tumors (RECIST) Version 1. 1. A CR was defined as no evidence of disease and tumor marker measurements within normal ranges for at least 2 weeks. Partial response (PR) was defined as a decrease in the lesion as measured bidimensionally by at least 30% with no signs of either new lesions or progression of any existing lesions. Progressive disease (PD) was defined as an increase of at least 20% in a lesion as measured bidimensionally, the appearance of any new lesions, or a previously eradicated lesion reappearing. Stable disease (SD) was defined as a tumor response that did not fulfill the PR criteria but exceeded the PD criteria. A primary tumor response that fulfilled the CR criteria and PR criteria was defined as objective response (OR) (OR = CR + PR), and the other was defined as Non-OR.

### PET/CT scanning and image analysis

PET/CT scanning was performed before any anti-cancer treatment with an advanced PET/CT scanner (Discovery LS, GE Healthcare). Before undergoing PET/CT scans, all patients were asked to fast for at least 6 h and have a blood glucose level ≤ 11.1 mmol/L. Then each of they were injected into 5.18 MBq/kg of ^18^F-FDG.One hour later, a whole-body PET and CT scans from top of the skull to the proximal thigh were initiated for 5 min per field of view, each covering 14.5 cm, with an axial sampling at 4.25 mm per slice. Then use the ordered-subset expectation maximization algorithm to reconstructed PET data sets by CT-derived attenuation correction. The attenuation-corrected PET images, CT images, as well as fused PET/CT images were displayed as coronal, sagittal, and axial slices on the Xeleris workstation (GE Healthcare). Measurements were obtained by two nuclear medicine physicians with at least 10 of experience and who were unaware of the patients’ clinical and pathological results. The standard uptake values (SUVs) were obtained with the contour threshold method and were based on a region of interest (ROI). An SUV threshold of 2.5 was used to define the ROI boundaries, which has been widely approved. A volumetric ROI was placed around the outline of the primary tumor on the axial PET/CT images using semiautomatic software. The ROI borders were manually adjusted by visual inspection of the primary tumor to avoid overlapping with adjacent FDG-avid structures. The SUVmean value is the average of the metabolic activity in the ROIs and was automatically calculated by the software. Metabolic tumor volume (MTV) was defined as the volume of the part of the primary lesion that was obtained using the cutoff (SUV ≥ 2.5). Total lesion glycolysis (TLG) was calculated by multiplying SUVmean by MTV.

### Laboratory data

Venous blood samples were collected between 6 and 9 am 1 week before any anti-cancer treatment, and both the peripheral neutrophils and lymphocytes were counted by Sysmex XT-2000i Automated Hematology Analyzer (GMI, MN, USA). The peripheral NLR was defined as the absolute neutrophil count divided by the absolute lymphocyte count.

### Statistical analysis

The selection of cut-off values for the baseline SUVmean and peripheral NLR was determined using receiver operating characteristic (ROC) curve analysis in the training set (*n* = 80). The correlations between SUVmean and the NLR and clinicopathological parameters were assessed by Fisher’s exact or chi-squared tests in the training set. Spearman’s correlation coefficient test was used to estimate the correlation between the SUVmean and NLR level. Univariate and multivariate logistic regression analyses were performed in the training set to identify the independent predictors for tumor response. The independent predictive factors were used to establish the prediction model for treatment response to CCRT in esophageal cancer patients and to construct the regression equation for calculating the model prediction value (Y-value). Delong’s test was used to analyze the area under the curve (AUC) of the ROC curves and to compare the accuracy of each prediction index (Y-value, SUVmean, and NLR). The Y-value of each patient was calculated in the testing set by using the regression equation. The sensitivity and specificity of the predictive model were evaluated in the testing set and in all patients. SPSS 22.0 program (SPSS Inc., Chicago, IL, USA) and MedCalc program (Version 18.11) were used to conduct these analyses, a two-sided *p*-value less than 0.05 was considered statistical significance.

## Results

### Patient characteristics

A total of 163 newly diagnosed ESCC patients treated with CCRT were retrospectively analyzed, including 127 (77.9%) males and 36 (22.1%) females. The median age was 65 years (range: 39–90 years). In our sample, there were slightly more patients who had a history of smoking (56.4%) than those who had never smoked; a similar distribution was observed for patients with a history of alcohol consumption. Of the 163 patients with ESCC, 12 (7.4%) tumors were located in the cervical, 50 (30.7%) were located in the upper thoracic, 75 (46.0%) were located in the mid-thoracic, and 26 (16.0%) were located in the lower thoracic esophagus. Additionally, most of the patients had stage III disease (122, 74.8%), whereas 41 (25.2%) had stage II disease. 112 (68.7%) patients in the OR and 51 (31.3%) non-OR groups were and, respectively, with an overall ORR of 68.7% (Table 1).

**Table 1:** Baseline clinical characteristics of patients

Characteristics	All cases (*n* = 163)	Training set (*n* = 80)	Testing set (*n* = 83)
**Age (years)**			
median	65	66	65
range	39–90	39–90	44–84
**Sex**			
Male	127 (77.9)	58	69
Female	36 (22.1)	22	14
**Smoking history**			
Yes	92 (56.4)	41	51
No	71 (43.6)	39	32
**Drinking history**			
Yes	86 (56.4)	41	45
No	77 (43.6)	39	38
**T stage**			
1–3	128 (78.5)	63	65
4	35 (21.5)	17	18
**N stage**			
0	137 (84.0)	63	74
1–3	26 (16.0)	17	9
**Tumor stage**			
II	41 (25.2)	20	21
III	122 (74.8)	60	62
**Tumor location**			
Cervical	12 (7.4)	5	7
Upper thoracic	50 (30.7)	27	23
Mid-thoracic	75 (46.0)	36	39
Lower thoracic	26 (16.0)	12	14
**Tumor response**			
OR	112 (68.7)	56	56
Non-OR	51 (31.3)	24	27

### Comparison of PET parameters

**Fig. 1:**
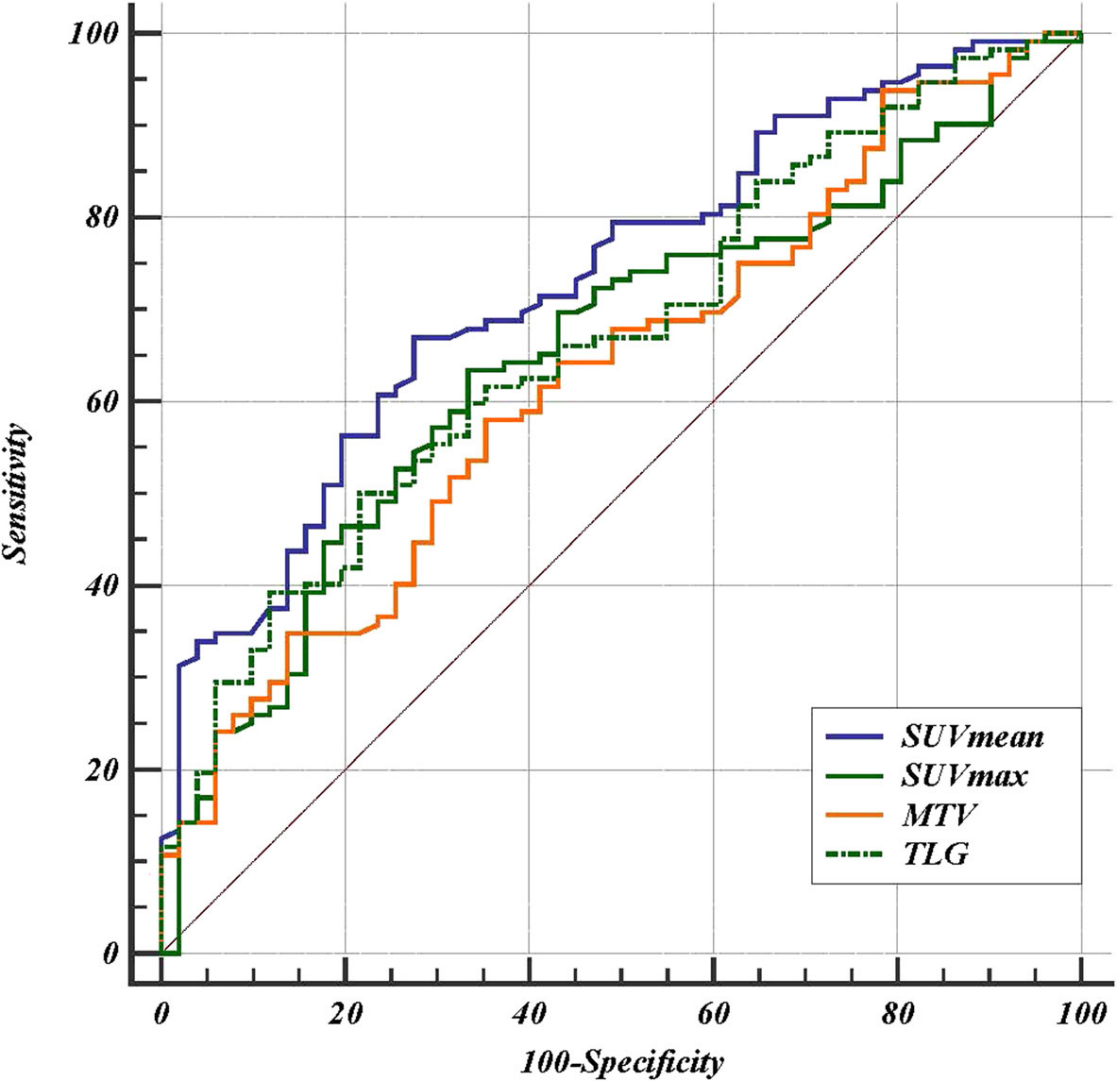
ROC curves of PET parameters

We conducted a preliminary analysis of the predictive effect of PET parameters on predicting the treatment response of CCRT in esophageal cancer by performing a receiver operating characteristic (ROC) curve. The results show that SUVmean has the highest predicting accuracy. The AUCs of ROC curve of SUVmean, NLR, MTV and TLG were 0.732 (95% CI: 0.657–0.798),0.652 (95% CI: 0.574–0.725),0.628 (95% CI: 0.549–0.703), and 0.668 (95% CI: 0.590–0.739), respectively (supplement Table 1). The ΔAUC (calculated by subtracting the AUCs of the other three PET parameters from that of SUVmean respectively) between SUVmax, MTV and SUVmean were 0.0797 and 0.1030, the difference was statistically significant (*p* = 0.0019, *p* = 0.0021, respectively). The ΔAUC between TLG and SUVmean has differential tendencies, but no statistical significance (ΔAUC = 0.0642, *p* = 0.00746) (Fig. 1).

### Univariate and multivariate analyses in the training set

**Fig. 2:**
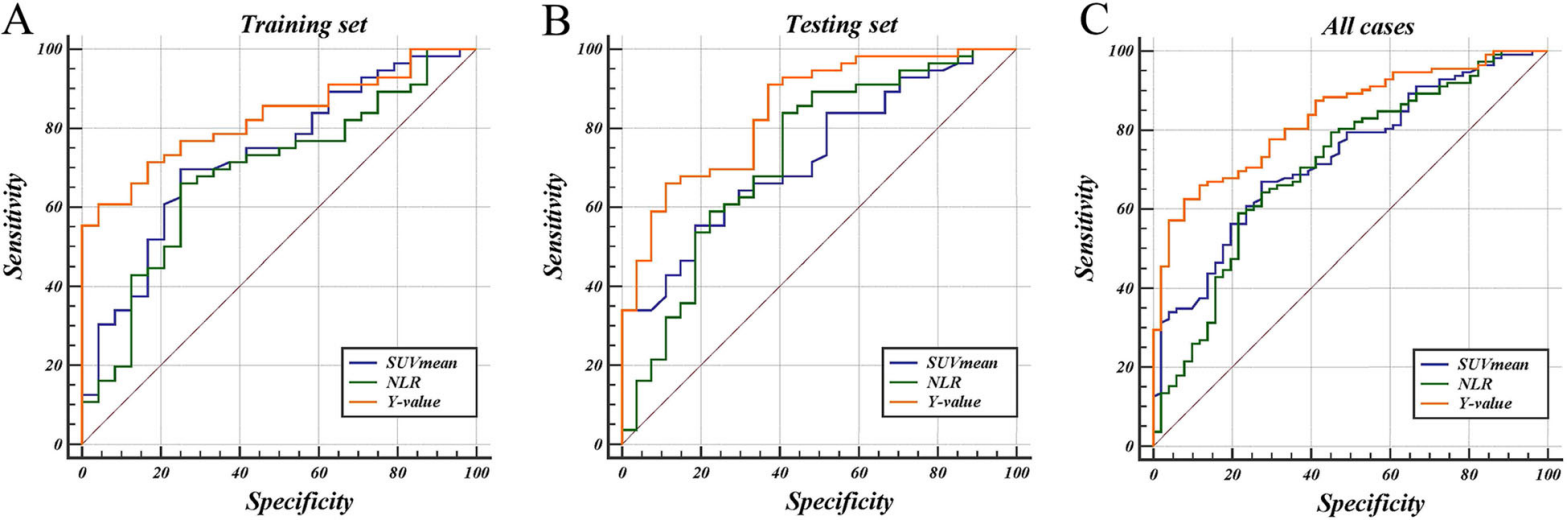
ROC curves of three variables for treatment prediction in different set samples

**Fig. 3:**
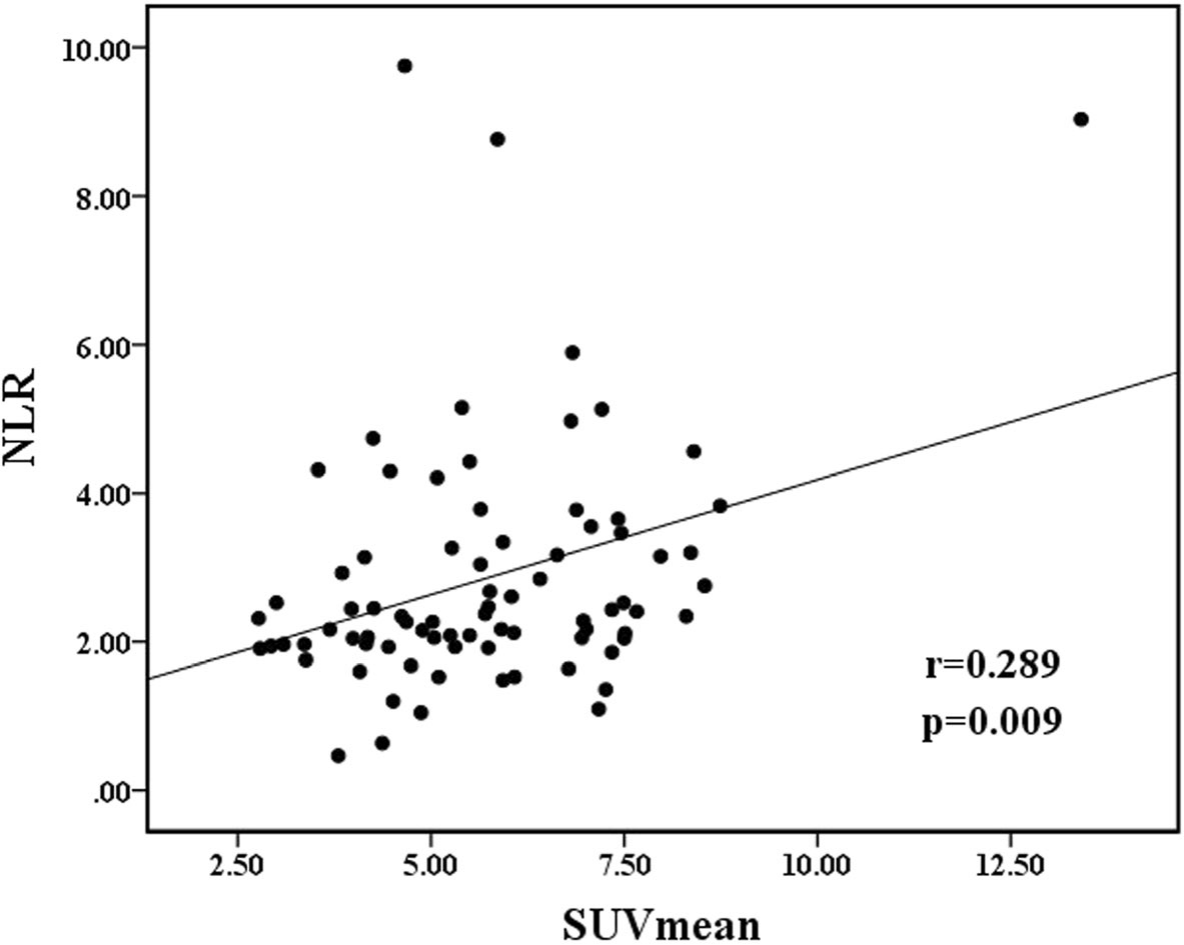
Spearman’s correlation analyses indicating a significant correlation between that SUVmean and NLR (r = 0.289, *p* = 0.009)

To understand the prognostic values of SUVmean and NLR, we determined the optimal cut-off values by receiver operating characteristic (ROC) curve analysis in the training set. The AUCs of the SUVmean and NLR were 0.731 (95% CI: 0.620–0.824) and 0.686 (95% CI: 0.573–0.785) with optimal cut-off values of 5.81 (sensitivity: 75.0%, specificity: 69.6%) and 2.42 (sensitivity: 75.0%, specificity: 66.1%), respectively (Fig. 2a). The baseline data of the patients in different SUVmean and NLR groups are summarized in Table 2. We examined the relationship between SUVmean and NLR. The result showed that there was a significant, however, quite weak positive correlation between SUVmean and NLR (r = 0.289, *P* = 0.009; Fig. 3). The univariate analysis revealed that tumor stage (*P* = 0.020), SUVmean (*P* < 0.001) and NLR (*P* = 0.001) were prognostic factors for OR. None of the other parameters (i.e., age, sex, smoking history, drinking history, T stage, N stage, and tumor location) showed significant differences among groups (Table 3). Subsequently, the multivariate analysis revealed that a low tumor stage (HR = 10.92; 95% CI, 1.17–102.12; *P* = 0.036), low NLR (HR = 7.17; 95% CI: 2.12–24.20), and low tumor SUVmean (HR = 3.95; 95% CI, 1.16–13.47; *P* = 0.028) were significant independent predictors for good treatment response.

**Table 2:** Baseline data between different SUV_mean_ and NLR groups in training set

Characteristics	SUVmean			NLR		
	≤5.81	> 5.81	*p*	≤ 2.42	> 2.42	*p*
**Age (years)**						
< 60	19	8	0.069	17	10	0.238
≥ 60	26	27		26	27	
**Sex**						
Male	32	26	0.752	30	28	0.555
Female	13	9		13	9	
**Smoking history**						
Yes	22	19	0.632	22	19	0.987
No	23	16		21	18	
**Drinking history**						
Yes	23	18	0.978	23	19	0.666
No	22	17		20	18	
**T stage**						
1–3	36	27	0.757	32	31	0.307
4	9	8		11	6	
**N stage**						
0	13	4	0.058	10	7	**0.636**
1–3	32	31		33	30	
**Tumor stage**						
II	18	2	**< 0.001**	10	10	0.698
III	27	33		33	27	
**Tumor location**						
Cervical	3	2	0.746	4	1	0.323
Upper thoracic	16	11		15	12	
Mid-thoracic	18	18		20	16	
Lower thoracic	8	4		4	8	
**Tumor response**						
OR	39	17	**< 0.001**	6	18	0.001
Non-OR	6	18		37	19	
**SUVmean**						
≤ 5.81	–	–	–	28	17	0.085
> 5.81	–	–		15	20	
**NLR**						
≤ 2.42	17	20	0.85	–	–	–
> 2.42	28	15		–	–

**Table 3:** Univariate and multivariate analyses for tumor response in training set (OR and non-OR)

variable	Categories	Univariate analysis			Multivariate analysis		
		HR	95% CI	*p*	HR	95% CI	*p*
**Age**	< 60 versus ≥60	0.556	0.190–1.621	0.282			
**Sex**	Male versus Female	0.833	0.280–2.480	0.743			
**Smoking history**	Yes versus No	1.074	0.413–2.795	0.884			
**Drinking history**	Yes versus No	1.074	0.413–2.795	0.884			
**T stage**	T1–3 versus T4	0.733	0.236–2.282	0.592			
**N stage**	T0 versus T1–3	0.273	0.057–1.309	0.105			
**Tumor stage**	II versus III	11.81	1.48–94.27	**0.020**	10.92	1.17–102.12	**0.036**
**Tumor location**	Cervical	2.000	0.201–19.914	0.554			
	Upper thoracic	1.429	0.473–4.313	0.527			
	Mid-thoracic	reference	–	–			
	Lower thoracic	1.000	0.250–3.998	1.000			
**NLR**	≤ 2.42 versus > 2.42	5.84	1.99–17.15	**0.001**	7.17	2.12–24.20	**0.002**
**SUVmean**	≤5.81 versus > 5.81	6.88	2.33–20.38	**< 0.001**	3.95	1.16–13.47	**0.028**

### Construction and validation of the prediction model

**Fig. 4:**
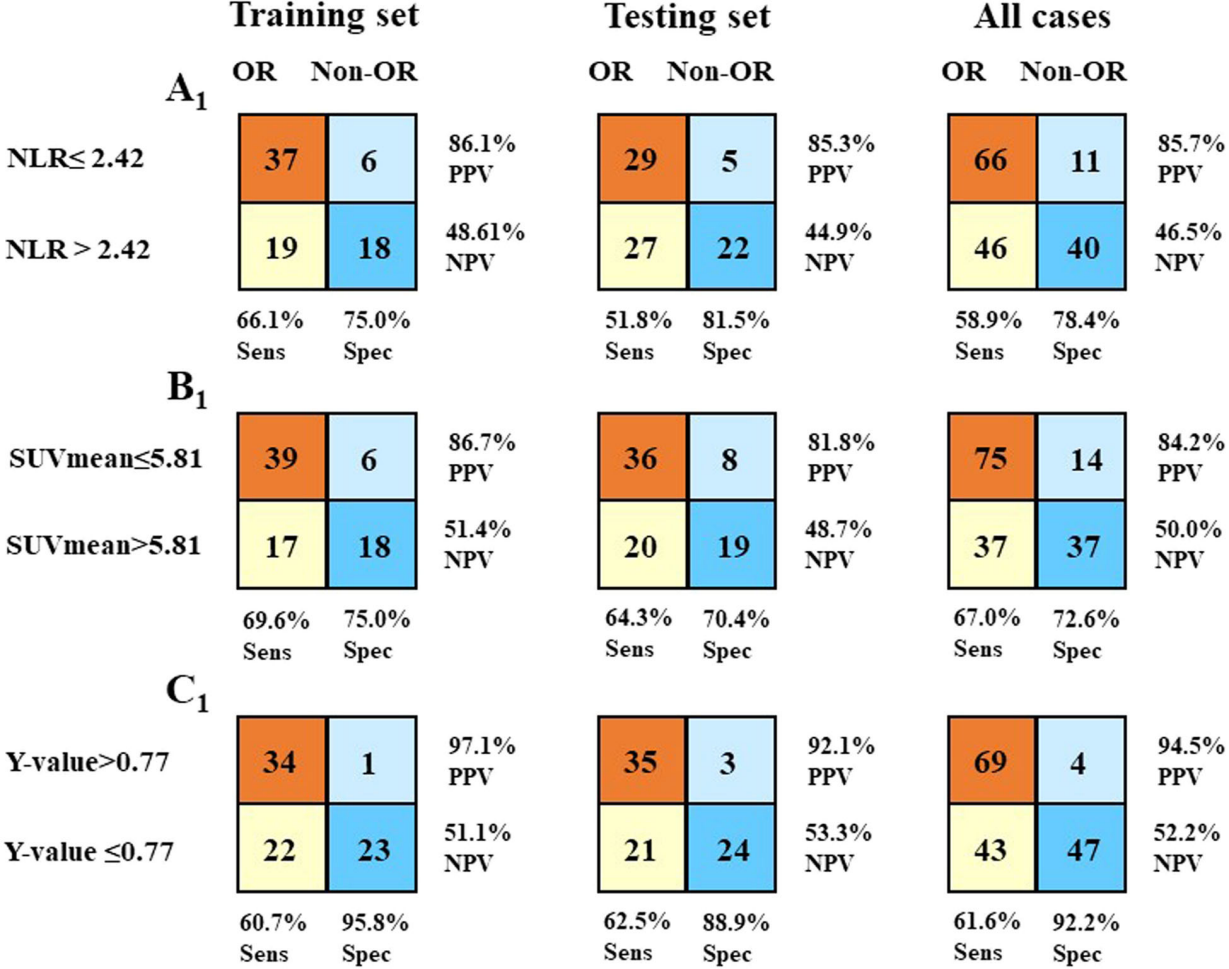
Performance of three variables in different set samples. For each variable, the sensitivity (Sens), specificity (Spec), positive predictive value (PPV), and the negative predictive value (NPV) are shown. (**a**): NLR; (**b**): SUVmean; (**c**): Y-value

**Fig. 5:**
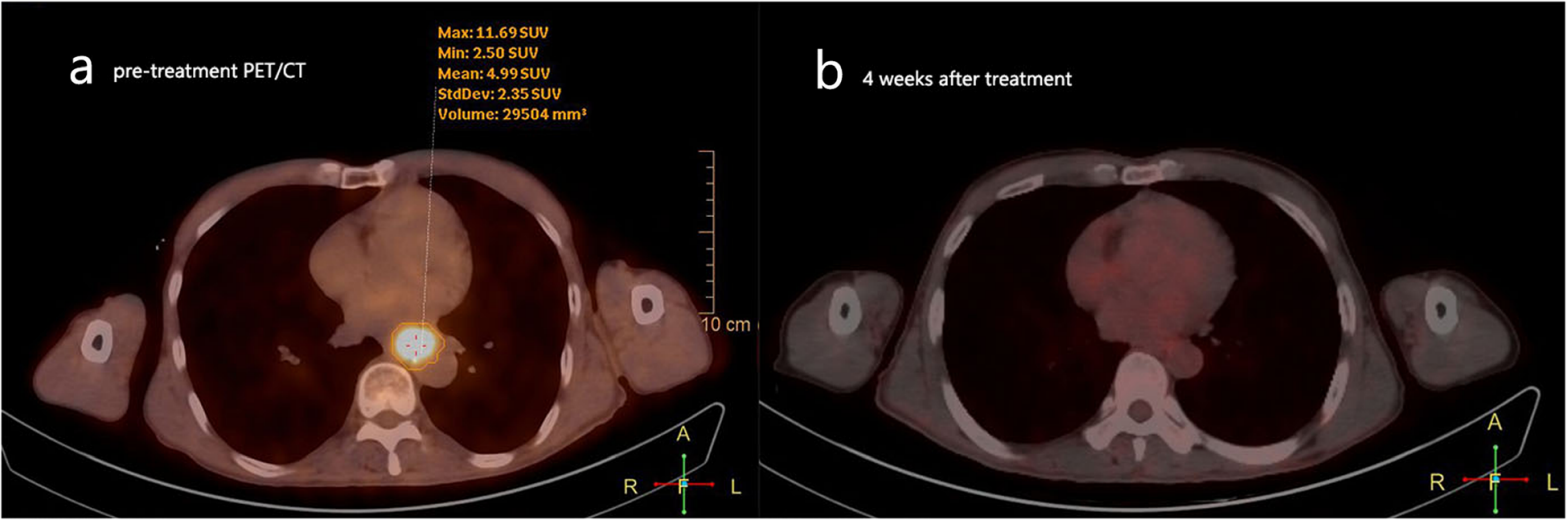
This is a stage III ESCC patient with a SUVmean value at 4.99 and the NLR value at 0.80. According to the prediction model, the patient has Y-value of 0.86, which belongs to a good response patient. After receiving CCRT and sequential 2 cycles of chemotherapy, the repeated PET/CT examination showed that the primary esophageal lesion completely disappeared, and the treatment response was CR according to RECIST (version 1.1) evaluation standard

Based on the multivariate analysis of the training set, a logistic regression model was generated using tumor stage, the SUVmean value and the NLR value. The analysis results of the model are shown in Table 4. The logistic regression equation of the prediction model is as follows: $\mathrm{Y}=1/\left[1+{\mathrm{e}}^{-\left(7.849-3.728\ast {\mathrm{x}}_1-0.449\ast {\mathrm{x}}_2-0.338\ast {\mathrm{x}}_3\right)}\right]$. In the formula, x_1_ is the score of the tumor stage (stage II: 0; stage III: 1), x_2_ is the NLR value, and x_3_ is the SUVmean value. The Y-values of each patient were calculated according to the predictive model equation. ROC analysis was then performed to validate the final model (including tumor stage, SUVmean, NLR) in the training set; the resulting AUC was 0.826 (95% CI: 0.725–0.902) with a cut-off value of 0.77. Comparing the AUCs of the predictive model with that of the “single model” (SUVmean or NLR) by Delong’s test, a significant *P*-value was observed (*P* = 0.048, and *P* = 0.012, respectively) (Fig. 2 a). The same procedure was then performed in the testing set and in all patients, and the results show that the full model also had a better performance than the “simple model” in both samples (Table 5). Subsequently, the patients in the testing set and all sets were dichotomized based on the cut-off values of the SUVmean, NLR and Y-values obtained in the training set. Each of the variables was then used to predict the treatment response of the three samples. The sensitivity, specificity, positive predictive value, and negative predictive value of each variable for predicting treatment outcomes were evaluated (Fig. 4). With a cut-off value of 0.77, the combined model significantly improved the specificity and positive predictive value. The specificity and positive predictive value increased to 95.8 and 97.1% in the training set, to 88.9 and 92.1% in the testing set and to 92.2 and 94.5% in all sets, respectively. However, the sensitivity and negative predictive value of the combined model did not seem to be better than those of the SUVmean or NLR values were. Figure 5 was a typical case presentation with PET image parameter.

**Table 4:** Logistic regression models fitted on training set samples

Variable	B	S.E.	Wald	df	p	OR	95%CI
**Tumor stage**	−3.728	1.873	3.960	1	0.047	0.024	0.001–0.945
**NLR**	−0.449	0.209	4.596	1	0.032	0.638	0.423–0.962
**SUVmean**	−0.338	0.194	4.008	1	0.045	0.678	0.464–0.992

**Table 5:** Comparison of ROC curves

Variable	AUC	SE.	95% CI	^a^AUC	*p*-value
**Training set**					
SUVmean	0.731	0.0608	0.620–0.824	0.0952	**0.0485**
NLR	0.686	0.0649	0.573–0.785	0.1400	**0.0122**
Y-value	0.826	0.0449	0.725–0.902	–	–
**Testing set**					
SUVmean	0.728	0.0562	0.619–0.820	0.117	**0.0167**
NLR	0.730	0.0628	0.622–0.822	0.115	**0.0204**
Y-value	0.845	0.0443	0.749–0.915	–	–
**All case**					
SUVmean	0.732	0.0406	0.657–0.798	0.102	**0.0022**
NLR	0.711	0.0445	0.635–0.780	0.123	**0.0007**
Y-value	0.834	0.0315	0.768–0.887	–	–

Notes: ^a^AUC was calculated by subtracting the AUC of SUVmean or NLR from that of Y-value respectively

## Discussion

In the present study, we demonstrated that the pretreatment SUVmean and NLR were independent predictive factors of treatment response to CCRT in patients with locally advanced esophageal cancer. Moreover, we developed a novel predictive model based on the pretreatment SUVmean and NLR values. This model had a good performance and may serve as an accurate and convenient tool for predicting the treatment response of patients and could make contributions to improving treatment outcomes and prognoses. To the best of our knowledge, this is the first predictive model for treatment response to CCRT in patients with locally advanced esophageal cancer that takes into account both tumor metabolic activity and host immunity.

^18^F-FDG PET/CT, which reflects glucose metabolism, has been widely applied in the management of oncological patients. In addition to detecting the primary tumor, this imaging modality also plays an important role in treatment response prediction. The semiquantitative data derived from such imaging, such as SUVmax and SUVmean as well as MTV and TLG, have been used for tumor response prediction in various cancers, including EC. Recent studies have shown that SUVmean provides a better picture of whole-tumor metabolic activity than SUVmax, which may only represent the single pixel of greatest metabolic activity within a tumor [10–13]. For example, a previous retrospective study of locally advanced cervical cancer revealed that patients with high SUVmean values were associated with poor post-treatment responses to definitive chemoradiotherapy [12]. Our results similar with this finding; patients with a low SUVmean (≤5.81) are more likely to have a good tumor response than those with a high SUVmean (> 5.81). Our research suggests that SUVmean has higher diagnostic accuracy than SUVmax, MTV and TLG in predicting the response to treatment,it is an independent predictor of treatment response in locally advanced esophageal cancer patients treated with CCRT.

Cancer-related inflammation affects tumor proliferation and survival, angiogenesis, metastasis, and response to treatment [15–17]. Indeed, inflammation is now considered one of the hallmarks of cancer. The precise mechanism of these correlations is not yet clear, but there are some hypotheses on this issue. On the one hand, neutrophils contain and secrete a large number of inflammatory factors that directly contribute to tumor angiogenesis, vascular formation, growth and metastasis [15–17]. In addition, the circulating neutrophils could act as a surrogate for tumor-associated neutrophils, which act as adhesive adapters between circulating tumor cells and the metastatic target and play an important role in tumor angiogenesis and growth by secreting vascular endothelial growth factor and matrix metalloproteinase [15, 17]. On the other hand, lymphocytes possess an anti-tumor effect by inducing tumor cell apoptosis and mediating antibody-dependent cell-mediated cytotoxicity [22–24]. Moreover, memory T-cells are considered to have a crucial role in carcinogenesis [25]. Based on the contributions of inflammation to carcinogenesis and tumor progression, the prognostic value of NLR has been investigated in various types of cancers [18–20, 26–28]. All of the previous studies came to the conclusion that an elevated NLR is associated with poor outcomes. However, evidence for the prognostic role of NLR in esophageal cancer is relatively controversial. Kosumi K et al. [29] investigated the relationship between the preoperative NLR and prognosis in 238 patients with esophageal squamous cell carcinoma. The results showed that with a median of 1.94 as the cut-off value, the high-NLR group had a 3-year cancer-specific survival rate and 3-year survival rate of 81.1 and 82.3%, respectively, which were significantly higher than those in the low-NLR group (59.8 and 68.4%, respectively). A high preoperative NLR was significantly associated with short overall survival. Another study found that an elevated preoperative NLR (≥5. 0) level can be used as an independent prognostic indicator to predict recurrence and death after esophagectomy. The patients with elevated NLR levels had poor cancer-free survival and overall survival [18]. However, on the contrary, some investigators have documented that the pretreatment NLR did not predict the outcomes of patients treated with esophagectomy [30, 31]. These studies focused primarily on the long-term survival of patients undergoing surgery for esophageal cancer, and the NLR cut-off values have not yet been fixed, varying from 1.95 to 5.0. The predictive value of NLR for treatment outcomes in patients with locally advanced esophageal cancer receiving CCRT has rarely been reported. Yoo EJ et al. retrospectively analyzed 138 patients with locally advanced esophageal cancer and concluded that an elevated NLR was an independent predictor of poor outcomes for patients treated with CCRT [20]. This result is similar to our study. In our study, we use a ROC curve to determine the cut-off value of NLR, which balanced sensitivity and specificity. The results of this study indicate that NLR is an independent predictor of treatment response in patients undergoing CCRT and that patients with a high NLR (> 2.42) are more likely to have a poor treatment outcome than patients with a low NLR. The similarities of our studies stress the importance of further research on NLR for predicting the treatment outcomes of CCRT.

PET parameters represent an estimate of glucose metabolism in the entire tumor lesion, and hematological inflammation parameters reflect the host’s anti-tumor immunological response. The combined evaluation of these two factors may provide complementary information and may be highly effective for predicting the outcomes and prognosis of patients. There are some previous reports that identified the relationship between PET parameters and hematological inflammation parameters. For example, Fujii T et al. showed a significant positive correlation between the NLR and SUVmax values in 143 patients with invasive ductal breast cancer [32]. A similar study conducted by Jeong E et al. [33] with 1034 newly diagnosed non-small-cell lung cancer patients investigated the relationship between SUVmax and circulating blood cell-based parameters. A weak but statistically significant correlation was found between SUVmax and NLR. Furthermore, several studies have demonstrated a direct association between metabolic tumor volume (MTV) and NLR [34–36]. In our present study, we determined that SUVmean also had a positive correlation with NLR. This result was consistent with previously reported findings [37]. However, the precise mechanism behind these correlations is complicated and is currently under investigation, but certain opinions may be useful for interpreting the mechanism. One possible opinion may be that inflammatory cells, such as lymphocytes, neutrophils, and macrophages, infiltrate the malignant lesions to increase the intake of FDG to reflect more energy consumption [38]. Another potential explanation may involve inflammation-induced angiogenesis. Hypoxia and persistent neovascularization are core features of the tumor microenvironment. Hypoxia in the tumor microenvironment promotes the secretion of angiogenic factors by increasing the number of inflammatory cells, resulting in the production of a large number of new blood vessels, which is then accompanied by an increase in tumor FDG uptake [39, 40]. These insights shed new insights into the relationship between tumor metabolic activity and the host’s inflammatory response process. The combination of these two types of parameters may serve as an effective predictor of treatment outcomes and prognoses. However, to date, we found only a few publications on this topic with cancer types such as intrahepatic cholangiocarcinoma [41], pancreatic cancer [42] and non-small cell lung cancer [43]. In the study of intrahepatic cholangiocarcinoma, researchers have developed a prognostic scoring system combining tumor SUVmax and NLR. The researchers assigned a prognostic score of 0 for patients with both low SUVmax and low NLR values, a score of 2 for patients with both high SUVmax and high NLR, and a score of 1 for the other patients. The researchers found significant differences in OS according to the prognostic scores. Similarly, the data from Shi S et al. [42] and St-Pierre Y et al. [43] proved that scoring systems that consider both metabolism parameters and inflammation parameters are able to stratify patients into different subgroups and are able to predict patient prognosis based on different scores. Although these studies demonstrate the predictive value of the combination of these two types of parameters, there were certain shortcomings in these studies. First, all of these studies simply scored patients as 0 or 1 based on the cut-off values of the metabolic and inflammatory indicators. These systems do not weigh the contribution of different indicators in predicting efficacy, which may lead to exaggerating or narrowing the role of a certain indicator. In addition, these systems do not include other factors that may affect prognosis. Second, these systems do not compare the performance of the scoring system with that of single indicators. In our study, we established a predictive model for treatment outcomes based on SUVmean and NLR in the training set that not only considers the contribution of different indicators but also includes other indicators that affect efficacy, i.e., tumor stage. Importantly, we verified the predictive performance of the model in the testing set and in all patients. Our data suggest that the accuracy of the prediction model is significantly better than that of the single SUVmean value or NLR value. With a cut-off value of 0.77, the model has a high specificity and positive predictive value for predicting the treatment outcomes of EC patients treated with CCRT, although the model did not show an advantage in terms of sensitivity and negative predictive value. This model might therefore be able to identify patients who may be highly sensitive to CCRT and thus give these patients treatment with an appropriate intensity to avoid unnecessary adverse reactions. For patients who are not sensitive to CCRT, their treatment intensity and type of treatment may need to be tailored before treatment, thereby improving their underlying poor response to treatment.

Several limitations in the present study should to be mentioned. The first is its retrospective nature. And it is a single center, small sample study. There are potential confounding factors that we cannot control. In the future, further prospective research should be conducted. Second, we do not have a clear explanation for the precise mechanism of the correlation between SUVmean and NLR. Finally, although we have demonstrated that this predictive model has a good performance in the testing set and in all patients, the model still needs to be verified by clinicians in practical work.

## Conclusion

The pretreatment SUVmean and NLR values were independent predictors of treatment response to CCRT in patients with esophageal squamous cell carcinoma. The predictive model, which was constructed based on the pretreatment SUVmean and NLR values, provides a highly accurate tool for predicting patient outcomes after CCRT. This model may help clinicians identify subgroups of patients who are sensitive or insensitive to CCRT and to give these patients individualized and accurate treatment.

## Supplementary information

**Additional file 1: Supplement Table 1.** AUCs comparison of PET parameters

## Data Availability

The datasets used and/or analyzed during the current study are available from the corresponding author on reasonable request.

## Abbreviations

^18^F-FDG PET/CT: ^18^F-fluorodeoxy-glucose positron emission tomography/computed tomography
SUVmax: Maximum standardized uptake value
SUVmean: Mean standardized uptake value
NLR: Neutrophil to lymphocyte ratio
CCRT: Concurrent chemoradiotherapy
ROC: Receiver operating characteristic
AUC: Area under the curve
HR: Hazard ratio
ESCC: Esophageal squamous cell carcinoma
EC: Esophageal cancer
ECOG: Eastern Cooperative Oncology Group
IMRT: Intensity-modulated radiation therapy
RECIST: Response based on evaluation criteria in solid tumors
CR: Complete response
PR: Partial response
SD: Stable disease
PD: Progressive disease
OR: Objective response
ORR: Overall response rate
