# Supplementary material for: A predictive model for treatment response in patients with locally advanced esophageal squamous cell carcinoma after concurrent chemoradiotherapy: based on SUVmean and NLR
Source: BMC Cancer. 2020 Jun 10;20:544. doi: 10.1186/s12885-020-07040-8 (PMC7288413; doi:10.1186/s12885-020-07040-8)
Supplement: Supplementary file 1 — Additional file 1: Supplement Table 1. AUCs comparison of PET parameters [file 12885_2020_7040_MOESM1_ESM.docx]

Supplement table 1 AUCs comparison of PET parameters

| **Variable** | AUC | SE. | 95% CI | ΔAUC | p |
| --- | --- | --- | --- | --- | --- |
| SUVmean | 0.732 | 0.0406 | 0.657-0.798 | reference | - |
| SUVmax | 0.652 | 0.0449 | 0.574-0.725 | 0.0797 | **0.0019** |
| MTV | 0.628 | 0.0458 | 0.549-0.703 | 0.1030 | **0.0021** |
| TLG | 0.668 | 0.0440 | 0.590-0.739 | 0.0642 | 0.0746 |
